# Bidirectional learning opportunities: How GP‐supervisors and trainees exchange knowledge

**DOI:** 10.1111/medu.14590

**Published:** 2021-07-16

**Authors:** Lisanne S. Welink, Tessa C. van Charldorp, Laura Di Colandrea, Marie‐Louise L. Bartelink, Peter Pype, Roger A.M.J. Damoiseaux, Esther de Groot

**Affiliations:** ^1^ Julius Center for Health Sciences and Primary Care University Medical Center Utrecht Utrecht University Universiteitsweg, Utrecht The Netherlands; ^2^ Faculty of Humanities Utrecht University Utrecht The Netherlands; ^3^ Department of Public Health and Primary Care Ghent University Ghent Belgium

## Abstract

**Introduction:**

Workplace‐based learning conversations can be a good opportunity for supervisors and trainees to learn from each other. When both professionals discuss their specific knowledge openly with each other, learning conversations may be a useful educational tool, for instance for learning how to apply evidence‐based medicine (EBM) in the workplace. We do, however, need a better understanding of how the exchange of knowledge provides opportunities for such bidirectional learning. The aim of this study was therefore to analyse how trainees and supervisors currently handle bidirectional learning opportunities by describing in detail how supervisors respond to knowledge expressed by trainees during a learning conversation.

**Method:**

We video‐recorded learning conversations between supervisors and trainees in general practice (GP). Within these learning conversations, EBM discussions on medical topics were selected and transcribed. We then identified, analysed using Conversation Analysis (CA) and categorised each expression of knowledge by the trainee and the supervisor's subsequent response.

**Results:**

We found that when a trainee expresses knowledge during the learning conversation, supervisors either (a) refute the expressed knowledge, (b) immediately suggest an alternative or (c) pose (additional) questions. These responses have consequences for the learning opportunities of both trainee and supervisor: it is only when supervisors pose further questions that trainees are encouraged to elaborate on their knowledge, leading to a bidirectional learning opportunity.

**Discussion:**

Improving EBM learning opportunities for both supervisors and trainees requires more than simply instructing trainees to express knowledge‐based—for instance—on recent evidence more often. Inflexible institutional roles related to historical claims of supervisors’ epistemic authority hamper bidirectional learning. Posing open questions during learning conversations enhances the flexibility of institutional roles while also creating bidirectional learning opportunities.

## Abstract 2

In workplace‐based conversations, supervisors can respond to trainees' utterances of knowledge by refuting, suggesting alternatives, or posing questions. Only the latter leads to bidirectional learning opportunities.

## 1 INTRODUCTION

Medical education relies a great deal on workplace‐based learning, in which trainees learn through, for instance ‘supported participation’ and dialogue.^1^ Dialogue between trainee and supervisor can be seen as one of the most important aspects of workplace‐based learning, being deliberative yet informal.^2^, ^3^, ^4^, ^5^ One specific workplace dialogue that could be a good learning opportunity for both supervisor and trainee is the ‘learning conversation’, a standard practice in general practitioner (GP) specialty training. Learning conversations are regularly scheduled meetings in which clinical supervisor and trainee discuss clinical questions, medical topics or personal development.^6^ They combine debriefing, supervision and feedback, in this way assigning the ‘learner’ role largely to the trainee while the supervisor advises, comments or instructs.^7^ Knowledge on how these dialogues precisely occur and how both trainee and supervisor can learn from exchanging knowledge during such dialogues is lacking.

Although workplace learning conversations are mainly seen as an opportunity for trainees to learn from supervisors, supervisors can also learn from trainees. Previous research on bidirectional learning opportunities showed that supervisors acknowledge that they could learn from their trainee during learning conversations.^8^, ^9^ Also research on collaborative learning has shown that learning between peers or professionals can lead to valuable learning outcomes due to the exchange of knowledge and perspectives.^10^, ^11^ However, collaborative learning is mostly researched in a setting of two peers. The concept of bidirectional learning, defined as reciprocal learning between supervisor and trainee, has gained surprisingly little attention within medical education research. So it remains unclear whether and how bidirectional learning opportunities during learning conversations are seized.

To analyse how bidirectional learning opportunities are approached, it is a good starting point to look at how trainees and supervisors learn to apply evidence‐based medicine (EBM). While traditionally, learning of EBM has been mainly focused at how to search and appraise clinical evidence, recent papers advocate for a more holistic approach in which the contextual application of EBM at the workplace is essential.^12^, ^13^ This involves learning how to take decisions about individual patients by combining (a) the best available evidence, (b) the patient's preferences and (c) the clinician's clinical expertise.^12^, ^14^, ^15^ However, combining all these relevant aspects during daily clinical practice is complex. When learning how to do this, trainees and supervisors may benefit from each other's strengths: trainees are likely to be more up‐to‐date on the latest evidence and have better literature search skills, while supervisors tend to know more about a patient's preferences and have more clinical experience. When both supervisor and trainee bring their specific knowledge to the table and discuss it openly with each other, bidirectional learning within learning conversations can be an especially valuable educational tool for learning how to apply evidence‐based medicine (EBM). Currently, it is not clear whether supervisor and trainee seize the opportunities to learn from each other during dialogues. In order to get a better view on the potential of bidirectional learning, it is essential to know how knowledge exchanges between supervisor and trainee currently occur.

To study bidirectional learning opportunities, we have applied the Conversation Analysis (CA) method. By analysing how people respond to each other's utterances, CA allows us to identify how people deal with asymmetries in knowledge, adding valuable new insights into learning and learning opportunities in medical education.^16^, ^17^, ^18^ By describing in detail how supervisors and trainees exchange EBM knowledge together during their conversations, this study aimed to analyse how trainees and supervisors currently approach bidirectional learning opportunities.

## 2 METHOD

### 2.1 Setting

In the Netherlands, postgraduate GP specialty training takes 3 years. In the first and last year, trainees work alongside an experienced GP, that is, their supervisor. One hour of the daily routine is set aside for workplace‐based learning conversations covering a range of topics, from medical cases in daily practice and training institute assignments to personal development. Trainees are expected to set the agenda for the conversation, but the supervisor can also add topics.

### 2.2 Data collection

Between September 2016 and April 2017, we used convenience sampling to select a heterogeneous group of nine established pairs of Dutch GP supervisors and trainees affiliated with the GP training institute in Utrecht, the Netherlands.^19^ The group differed in terms of the trainee's stage of training, the supervisor's age and experience, the length of collaboration between supervisor and trainee and the type of practice—solo, duo or health centre—in which they worked. Each pair was asked to video‐record two learning conversations, both of which had to include at least one discussion of a medical topic, since that is how we defined an EBM dialogue. Since Conversation Analysis focuses on naturally occurring talk and naturalistic data the pairs received as little guidance as possible on which conversations to record and on the purpose of the study.^20^ They were aware that the purpose of the study was to gain more insight on EBM learning at the workplace.

To ensure a diverse range of medical topics, we selected one discussion of a medical topic per learning conversation, producing a dataset of 18 medical discussions in total, which we transcribed verbatim. These discussions lasted between 5 and 20 minutes and showed how a trainee and a supervisor discussed a medical question. The discussions selected, for example involved discussions regarding the appropriate medication for benign prostatic hyperplasia (BPH) or protocols on administering vitamin D supplements to the elderly. The discussion started with the trainee asking a question or introducing the topic, followed by a dialogue on that question or topic, and concluded with a wrap‐up or transition to another topic. We examined our dataset of 18 medical discussions for opportunities for bidirectional learning. Since learning conversations are traditionally seen as an opportunity for trainees to learn from supervisors, we defined bidirectional learning opportunities as moments presenting learning opportunities not only for trainees but also for supervisors, with trainees expressing their knowledge to their supervisor.

### 2.3 Analytical procedure

**FIGURE 1:**
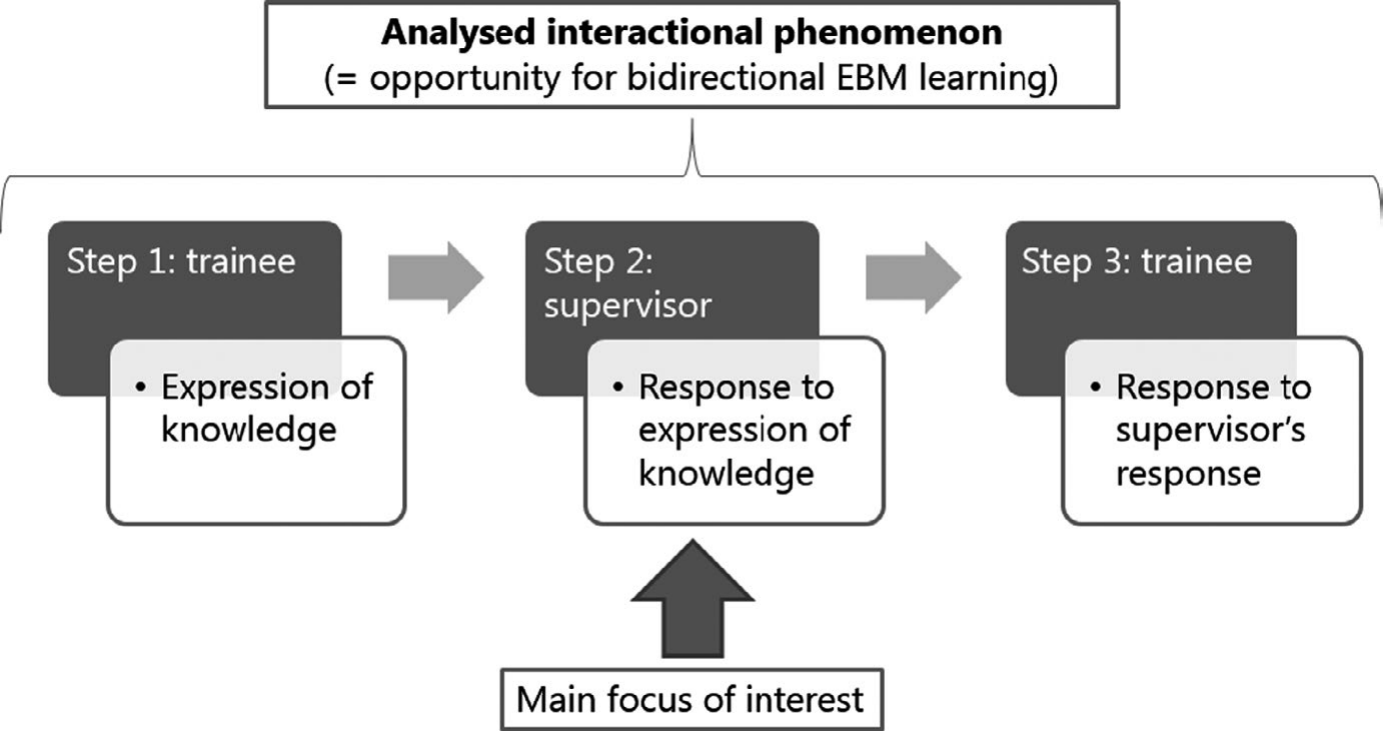
Illustration of the interactional phenomenon that was analysed, including relevant sequences

Since CA is an inductive method, we started by taking an open approach to our dataset, focusing on the utterances in which trainees expressed knowledge.^21^ We organised a data session in which ten CA researchers from various backgrounds commented on and analysed specific fragments, using the verbatim transcripts and anonymised video‐ and audio material.^22^ We were then able to describe our phenomenon of interest in greater detail. CA focuses on how a conversation unfolds, turn by turn, emphasising that language is co‐constructed and happens according to fixed patterns.^17^, ^20^, ^21^, ^23^ CA sees knowledge as a socially constructed process that occurs in interaction with others through mutual social actions.^20^, ^24^, ^25^, ^26^ Since CA looks at previous and subsequent turns in the interaction (*why that now* ^27^), we began our analysis by considering the trainee's expression of knowledge. We subsequently focused on how the supervisor responds to the trainee's expression of knowledge, as this provides interactional learning opportunities.^28^, ^29^, ^30^, ^31^ We also included the trainee's utterance following the supervisor's response. Figure 1 depicts the interactional phenomenon, including the three steps that we analysed.

We then selected one fragment from each supervisor‐trainee pair (nine in total) in which this interactional phenomenon was present. We transcribed these fragments in detail using Jeffersonian transcription conventions and analysed the fragments according to CA standards.^32^, ^33^ Individual case analysis was undertaken, with a focus on the design of individual turns at talk and the relationship between turns.^34^, ^35^ Grouping fragments that showed a similar response by the supervisor to the trainee's utterance of knowledge allowed us to form three sub‐collections.^20^, ^21^ We were thus able to identify patterns in how supervisors respond to an expression of knowledge by a trainee and whether this response promoted bidirectional learning opportunities.

To verify whether the identified patterns also applied to other sequences, we returned to the 18 medical discussions as a whole. Within these 18 discussions, two researchers (LW and LdC) looked for all sequences in which the trainee expressed knowledge. A total of 25 moments could be identified. All 25 moments fit within the three defined categories, leading us to conclude that the results and fragments presented in the Results section are illustrative of the 25 sequences in the complete dataset. The researchers discussed all analyses and conclusions at length and in detail within the research group.^36^

### 2.4 Ethical considerations

This study was part of a larger research project on EBM learning in the GP workplace. Approval for the research project as a whole, in which also Belgian GP supervisors and trainees took part, was granted by the ethical board of the NVMO (Dutch Society of Medical Education) under case number 706. Belgian ethical approval was issued by the Ethics Committee of the University Hospital of Ghent.

## 3 RESULTS

Three kinds of responses by the supervisor could be identified. Supervisors (a) refute the expressed knowledge, (b) immediately suggest an alternative or (c) pose (additional) questions. These three kinds of responses will be described below, using fragments that are illustrative of the interactional phenomena.

### 3.1 Refuting

The first response can be described as the supervisor refuting the expressed EBM knowledge **(**Box 1 **)**. In this fragment, the trainee and supervisor discuss the amount of levothyroxine that the trainee prescribed to a female patient. An explanation of the transcription symbols can be found in Table 1 below.

**TABLE 1:** Jeffersonian transcription conventions

The symbols listed below are based on Jefferson's glossary of transcript symbols, which are routinely used in conversation analytic transcription^32^	
Symbol	Definition
?	Strong rising phrase intonation
.	Strong falling phrase intonation
,	Slightly rising phrase intonation
;	Neutral phrase intonation
Q…Q	Indicates that the speaker reads something out loud or quotes
↑	Rise in intonation
((coughs))	Verbal description of (non‐verbal) actions
° okay.°	Softer than surrounding speech
:	Indication of a stretched sound
(.)	Pause or silence less than 0.2 seconds
(4.8)	Pause or silence of 4.8 seconds
=	Indicating there was no pause between the end of one utterance and the beginning of the next
>word<	Faster than surrounding speech
•h	Audible inhalation
[word]	Square brackets show where speech overlaps
(xx)	Inaudible speech
CAPITALS	Louder than surrounding speech

BOX 1  

1

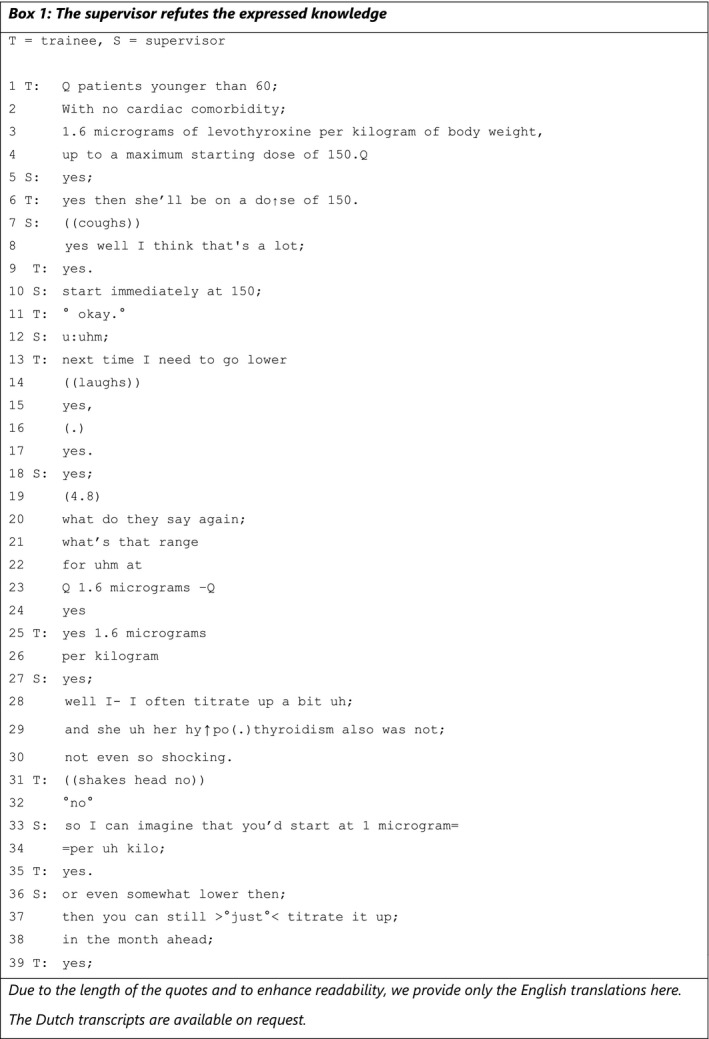

In lines 1‐6, the trainee expresses her knowledge by reading aloud the guideline to account for her deciding on a dosage of 150 (line 6). While the supervisor confirms the explicit reference to the guideline in line 5, the trainees’ conclusion in line 6 is refuted by the supervisor in line 8. This refutation is not directed to the specific, theoretical knowledge presented by the trainee in lines 1‐4, but refers to how to interpret and use this information in practice. The supervisor formulates his refutation in line 8 by starting with an agreement token (yes) and continuing with his own opinion, using the first‐person perspective (I) and the verb ‘think’ (‘I think that's a lot’), but without immediately explaining the source of his opinion. The addition of the word ‘well’ (line 8) illustrates a correction of the previous turns or information.^37^ Following this refutation, the trainee responds with an accepting ‘okay’ (line 11) and proceeds to draw a conclusion about what to do in any future encounter (line 13), in this way accepting the supervisor's refutation. The trainee gives a nervous laughter during this turn, demonstrating a tension or consciousness that the two ideas (supervisor's and trainee's) are not (as yet) aligned.^38^ Even though the trainee has already accepted his suggestion, the supervisor proceeds to substantiate his opinion by first referring to the guideline, using ‘they’ (‘what do they say again’, line 20) and adding a description of his own approach (line 28: ‘I often titrate up a bit’) and mentioning this patient's specific situation (lines 29‐30). The supervisor concludes by suggesting in lines 33‐34 and lines 36‐38 how to proceed. He does with the phrase ‘I can imagine’ (line 33), implying that his approach might be an option instead of an obligation, but, on the other hand, also guiding the trainee towards a preferred response in the next turn (‘yes’) (line 35).^39^ The trainee acknowledges these substantiations and suggestions multiple times with confirmatory responses such as ‘no’, ‘yes’ and ‘okay’ (eg lines 32, 35 and 39). When the supervisor immediately refutes the trainee's expressed knowledge, a discussion ensues in which the trainee no longer accounts for or expands on her knowledge and the supervisor does not ask for additional knowledge, missing an opportunity for bidirectional learning. The trainee accepts the supervisor's utterances without asking additional questions to improve her understanding, while the supervisor holds on to his ‘teacher’ role.

### 3.2 Immediately suggesting an alternative

Another way in which supervisors deal with trainees’ expressed knowledge is to simply ignore it and immediately suggest an alternative. A detailed example is provided in Box 2, in which supervisor and trainee discuss a case handled by the latter in which a female patient with Parkinson's disease has sleeping problems.

BOX 2

 

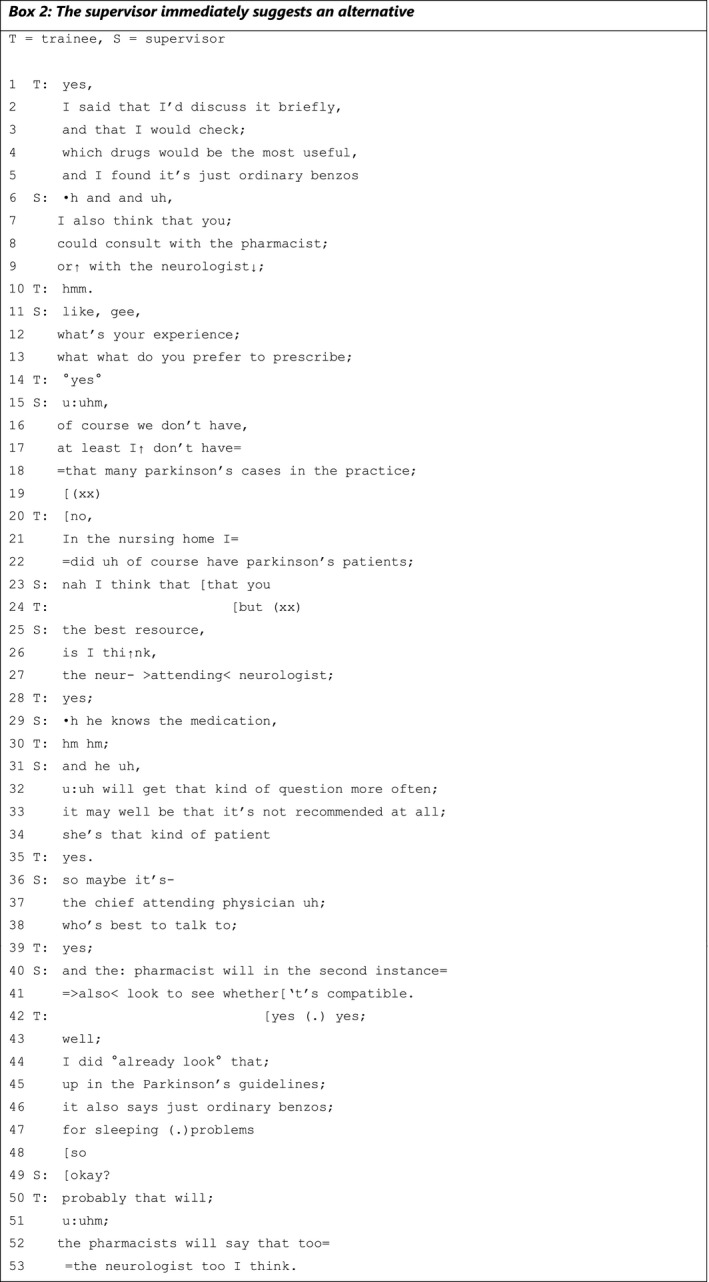

The knowledge expressed by the trainee is presented in lines 1‐5, where she concludes that, in this case, ‘benzos’ (line 5) are the best option, based on what she has ‘checked’. By using the term ‘just ordinary’ (line 5), she appears to be normalising her finding that this is the most suitable drug. In the next sequence, the supervisor does not respond to the substance of the trainee‘s knowledge expression but suggests an alternative. His and‐prefaced suggestion (‘and’ in line 6) and ‘I also think that you’ (line 7) implies actions above and beyond what the trainee has presented, in this way treating the trainee's previous turn not as a conclusion but as an option. He then continues by advising the trainee to consult an external source. Lines 15‐18 explain why he is advising the trainee to turn to external specialists: the supervisor himself lacks experience in this area (‘I don't have that many Parkinson's cases in the practice’). By adding ‘of course’, he implies that the absence of such patients is only logical. The supervisor's rephrasing of his statement to ‘at least I’ gives the trainee leeway to respond by recounting her own experience with Parkinson's patients (lines 21‐22). However, the supervisor once again does not respond to the trainee's experience but instead repeats his advice to consult the neurologist. He starts his turn with ‘nah’, which signals a contrasting or unexpected response to what has just been said.^40^ He elaborates on why this alternative seems best to him, referring to the neurologist's expertise (‘will get that kind of question more often’, line 32) and the patient specifics (‘she's that kind of patient’, line 34). While introducing his substantiation, the supervisor twice says ‘I think’ (line 23 and line 26), implying that he is not basing his recommendation on factual knowledge but on personal opinion or doubt.^41^ The trainee responds to his suggestions by referring to the external source again: she repeats that she has checked the specific guidelines (lines 43‐47) and it says ‘just ordinary benzos’. By bringing up the guideline, the trainee appears to be appealing to an external authority and thus minimising the disagreement.^42^ In the end, supervisor and trainee do not align; the trainee ultimately states that it's unnecessary to consult these specialists because they will reach the same conclusion. In this example, we see that ignoring the trainee's expressed knowledge by immediately suggesting alternatives prevents a knowledge exchange in which trainee and supervisor come to a shared decision or a general consensus on which knowledge is decisive, the medical guidelines or practical experience. They end up in a learning conversation in which they miss an opportunity for bidirectional learning.

### 3.3 Posing questions

Finally, a supervisor can respond to expressions of knowledge by posing additional questions that may have various different aims: (a) to clarify the expressed knowledge **(**Box 3 **)** or (b) to clarify the described situation **(**Box 4). In Box 3, supervisor and trainee discuss the case of a boy with an immune deficiency and persistent diarrhoea whose mother has celiac disease. The boy has already been tested for celiac disease and the result came back negative.

BOX 3

 

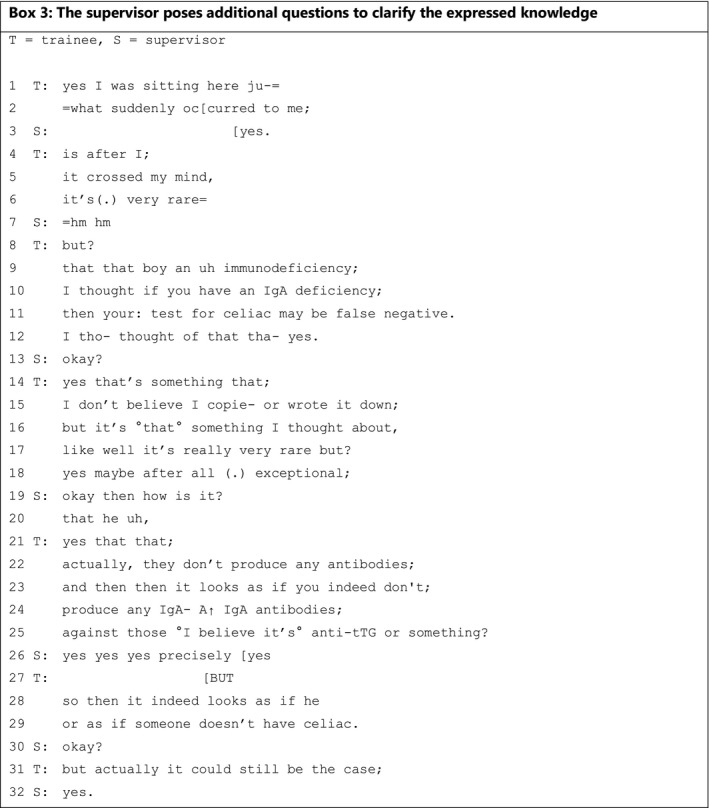

In lines 1‐12, the trainee is hesitant about presenting his knowledge and uses several utterances and linguistic markers to emphasise that it is only his thinking: he says that ‘it suddenly occurred to me’ (line 2), that ‘it crossed my mind’ (line 5), and repeats that it is something that he ‘thought’ (lines 10 and 12). Moreover, he adds that it is ‘very rare’ (line 6), which makes it safe for him to utter this possibility, implying that it might not be correct because it is rare but could be an option. The trainee does not present any explicit substantiation of the origin of this knowledge. The supervisor's primary response is to use continuers, such as ‘yes’ and ‘hm hm’, which leads the trainee to elaborate (lines 8‐12 and lines 14‐18).^43^ The trainee emphasises again that it is ‘very rare’ but that this case might still be an ‘exceptional’ one (line 18). The supervisor does not respond to the phenomenon possibly being ‘very rare’ but instead asks an additional question after the trainee's utterance that the test could be ‘false negative’ (line 11). The supervisor does this by asking for extra information on the origin of the possible ‘false negative’ outcome (lines 19‐20). By starting with the word ‘okay’, she seems to accept the presented knowledge, leading the trainee to elaborate more extensively in the lines 21‐31. The trainee starts his answer with the lexical usage of ‘actually’ (line 22), emphasising the utterance ‘they don't produce any antibodies’.^44^ With the addition of the words ‘or something’ in line 25, the trainee is once again creating leeway for mistakes or necessary adjustments in the presented knowledge, but this appears to be unnecessary given the supervisor's agreeing response in line 26. Without the supervisor explicitly requesting it, the trainee continues to elaborate in lines 27‐29 and 31. He does this with the word ‘but’, a cue that he is returning to the subject.^45^ In conclusion, posing additional questions invites the trainee to elaborate on the expressed knowledge, creating learning opportunities for both trainee and supervisor. This example shows that it is possible for supervisor and trainee to reach consensus on new knowledge expressed by the trainee (lines 27‐32).

The excerpt presented in Box 4 shows a supervisor asking a question to seek clarification of a specific case rather than to get the trainee to elaborate on (theoretical) knowledge. Supervisor and trainee discuss the risk of inducing hypotension when starting ACE inhibitors and diuretics at the same time to treat hypertension.

BOX 4

 

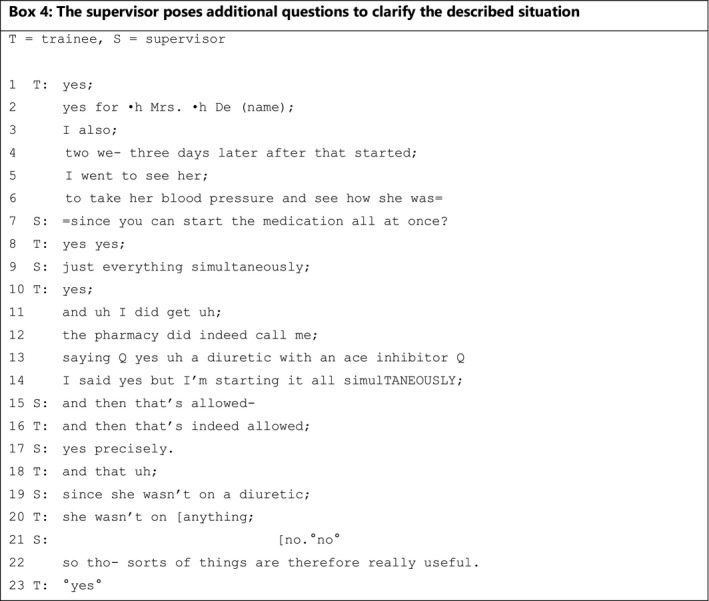

The trainee describes what he did during a recent case in lines 1‐6. In line 7, the supervisor utters a declarative question about starting the two kinds of medication ‘all at once’. In line 8, the trainee confirms that he was allowed to start these medicines ‘all at once’. Who allowed him to do so is not made clear. The supervisor rephrases the question in line 9, which the trainee once again confirms. The rephrasing of the question causes the trainee to substantiate his actions in more detail: in lines 11‐14 and 16, he explains that the pharmacy contacted him about starting ACE inhibitors and diuretics at the same time and allowed it in this specific situation. In contrast to Box 3, the supervisor then incorporates the situation of this specific patient lexically (‘she’) into a questioning utterance in which he seeks confirmation (line 19). Confirmation that the patient was not on any medication is given in line 20, again incorporating the lexical usage of ‘she’. By asking for clarification in lines 7, 9 and especially 19, the supervisor creates a learning opportunity for himself about how to combine knowledge and this patient's specific situation, since this invites the trainee to elaborate on and specify his knowledge.

## 4 DISCUSSION

This study illustrated how GP trainees and supervisors currently handle bidirectional learning opportunities during learning conversations by analysing in detail how supervisor and trainee together deal with the trainee's knowledge utterances. All the supervisors’ responses were found to have consequences for the learning conversation and, in turn, for bidirectional learning. It was only during conversations in which supervisors posed additional questions that trainees were encouraged to elaborate on their knowledge and a bidirectional learning opportunity emerged.

Our results add to current literature on dialogues at the workplace by providing real‐world insights to the reciprocal aspect of workplace‐based learning. No previous research has been done on how bidirectional learning takes place within clinical workplace‐based conversations. Previous CA‐research on workplace‐based dialogues focussed on the mechanisms of feedback, and thus, on the learning process of the trainee.^46^ Studies outside the medical field did look at the utterances that can support collaborative learning, for instance in collaborative writing of primary school children.^47^ Since no previous study looked in this much detail at the mutual construction of knowledge, these results aim to unravel a bit of the ‘butterfly effect’: the tiny variations and nuances that can lead to great effects within complex and dynamic systems like workplace‐based learning.^48^

Our results are related to perceptions of epistemic authority and show parallels with previous CA‐research in this field.^49^ Trainees easily position themselves in a subordinate epistemic position by spontaneously and extensively accounting for their knowledge, by referring to an external source as evidence, or by using epistemic hedges such as ‘I think’ or adverbs such as ‘probably’ (Box 2 and 3).^50^, ^51^, ^52^ On the other hand, supervisors maintain an epistemic authority position linguistically by, for instance normalising their own lack of knowledge or experience (Box 2, line 16: use of ‘of course’) or by recommending how to proceed without checking whether the trainee agrees (Box 1). In most institutional interactions, that is doctor‐patient interactions, classroom talk or mortgage consultations, the ‘institutional agent’ —the physician, teacher or mortgage advisor—conveys expert information to a patient, student or client.^23^, ^35^, ^53^, ^54^, ^55^, ^56^ In other words, participants are tied to their institution‐relevant identities.^57^ Likewise, the GP trainees and supervisors in this study maintain their traditional identities and interact according to the customary view of workplace‐based learning: supervisors present their knowledge or experience and teach trainees how to become good physicians, in this case GPs. Nevertheless, to optimise bidirectional learning, it is worth seeking out bidirectional learning opportunities and having both trainee and supervisor reflect on these interactional moments.

Bidirectional learning can occur when supervisors and trainees are able to make sense of clinical work together through dialogue and when they co‐construct their knowledge while involving contextual factors.^2^, ^10^, ^18^, ^58^, ^59^, ^60^ It appears that the most successful approach is when the supervisor poses additional questions regarding the knowledge expressed by the trainee. Displays of mutual understanding and indicators of successful learning were clearest when the supervisor posed additional questions (for instance in Box 4, line 17: ‘yes precisely’).^56^ Our results also show that these additional questions make institutional roles more flexible, creating learning opportunities for the supervisor as well.^41^, ^52^

## 5 STRENGTHS AND LIMITATIONS

This study uses Conversation Analysis to offer a fine‐grained examination of the learning opportunities that arise during conversations between trainees and supervisors in the workplace. As a data‐driven method with a basis in actual rather than idealised workplace conversations, the usage of this methodology can be seen as a strength. As Peräkylä (2003) aptly describes it, ‘*Theories and concepts related to practices consist of ideals and visions of the “best possible situations”, whereas institutional practices constantly deal with the range of cases that do not reach such ideals.’* ^61^ This also applies to EBM learning conversations, where clinicians often struggle with abstract instructions for applying the theoretical concept of EBM in daily clinical practice. The CA methodology helps to paint a more detailed picture of current practices and adds a new dimension to our understanding of these practices. Moreover, because the focus of CA is on collecting and analysing instances of real‐life interactional phenomena, the results of CA studies have ecological validity (ie the extent that the results are generalisable to real‐life settings). This means that the interactional phenomenon described in this paper may also be transferable to other settings, such as supervisor‐trainee discussions across the healthcare domain.

Since this study describes the consequences of particular utterances, it furthermore sheds light on how learners co‐regulate their EBM learning. Numerous studies focus on how learners handle their learning processes themselves, as forms of self‐regulated learning.^62^, ^63^, ^64^, ^65^, ^66^ This study, however, emphasises the role of co‐regulated learning, providing additional evidence that learning is embedded in social interactions between supervisor and trainee.^18^, ^58^

This study only looked at the opportunities for bidirectional learning during EBM‐related discussions within learning conversations. However, bidirectional learning might also take place during dialogues on different topics, such as communication or professional performance and attitude. It cannot be ruled out that supervisors respond differently to the trainee's utterances when the conversation concerns a different topic. Conversation Analysis with a more extensive dataset needs to be performed to give a complete overview of the ways that bidirectional learning opportunities are handled during learning conversations in general. While this study illustrates how current interactions between supervisor and trainee influence EBM learning opportunities during learning conversations, it remains unclear precisely how we should teach supervisors and trainees to optimise their interaction during such conversations. Posing open questions is of course a well‐known didactic tool, but simply advising professionals to pose open questions might not make institutional roles more flexible, so that bidirectional EBM learning is more successful. Further research on the Conversation Analytic Role‐play Method (CARM), for instance would promote progress in using learning conversations to enhance daily workplace‐based EBM learning.^67^

## 6 CONCLUSIONS AND RECOMMENDATIONS

Conversation Analysis of learning conversations between supervisors and trainees in general practice shows that bidirectional EBM learning opportunities are not always handled successfully. Improving EBM learning opportunities for both supervisors and trainees requires more than simply instructing trainees to express knowledge based—for instance —on recent evidence more often. Inflexible institutional roles related to traditions of epistemic authority hamper bidirectional learning. To make expressing knowledge appropriate for bidirectional learning, the supervisor and trainee must co‐construct learning opportunities and aim to make institutional roles more flexible by posing open questions about the expressed knowledge or situations.

## CONFLICTS OF INTEREST

The authors declare that they have no conflicts of interests.

## AUTHOR CONTRIBUTIONS

LW is a PhD candidate and former GP trainee. TvC is an assistant professor with a background in communication and linguistics, working as a researcher using CA. LDC is a graduate student in the field of communication and information sciences and a research assistant. MLB is an associate professor and a teacher in EBM. PP is an associate professor in General Practice and interprofessional collaboration. RD is a professor in General Practice and the head of GP specialty training. EdG is an assistant professor and researcher in the learning sciences in the medical domain.

## ETHICAL APPROVAL

Details on ethical approval are provided in the manuscript in the method section.
